# Arc Welding Processes as Practical Solutions to Join Ceramics: Progress and Future Outlook

**DOI:** 10.3390/ma18214940

**Published:** 2025-10-29

**Authors:** J. G. Lopes, J. Shen, J. P. Oliveira

**Affiliations:** CENIMAT/I3N, Department of Materials Science, NOVA School of Science and Technology, Universidade NOVA de Lisboa, 2829-516 Caparica, Portugal; j.shen@fct.unl.pt (J.S.); jp.oliveira@fct.unl.pt (J.P.O.)

**Keywords:** welding, ceramics, dissimilar welding, plasma arc welding, gas tungsten arc welding, fusion welding

## Abstract

Arc welding of ceramic materials remains a significantly underdeveloped research topic in materials science and manufacturing. This is primarily due to the difficulty in managing thermal gradients and stress concentrations during arc welding, which often result in cracking and poor joint reliability in ceramic components. However, recent advances suggest that gas tungsten arc welding and plasma arc welding could offer a promising pathway for ceramic joining if thoroughly investigated. While these techniques have proven effective in the precision welding of metals, their application to ceramics is met with several challenges, driven by the material’s inherent brittleness and high melting points. Consequently, further research is essential to understand the arc welding processes on ceramic materials and develop solutions to optimize and establish reliable welding procedures. Therefore, expanding this topic could have a significant impact on key engineering applications, where ceramic materials are increasingly in demand.

## 1. Introduction

Ceramics are non-metallic, inorganic materials that play a crucial role in modern industry due to their unique combination of properties. For example, they are valued for their high strength, heat and corrosion resistance [1,2], low density [3,4], and ability to function in extreme wear environments [5,6]. These properties make them essential for several industrial sectors, such as aerospace [7,8], automotive [9,10], electronics [11,12], energy storage [13,14], and construction [15,16].

Amongst the conventional processes utilized for producing ceramic-based components, such as sintering or hot pressing, the current literature [17,18] indicates that an effort to use well-established joining techniques is being made to enable the fabrication of complex ceramic structures without compromising their unique properties. In this context, advanced joining methods can be tailored to enable the reliable integration of ceramics into multi-material systems. This would not only facilitate repairs and reduce manufacturing costs but also enhance design flexibility.

Among the various joining methods, welding stands out as a broad category encompassing numerous techniques that offer a fast, effective, and often reliable way to create continuous joints. When guided by proper design specifications and process optimization, these processes have the potential to produce strong and durable bonds, classified according to the diverse mechanisms and procedures employed to achieve such joints.

In the first instance, there is solid-state joining of ceramics, which relies on diffusion mechanisms to generate a bond between the base materials (BMs). Such an approach is particularly valuable for ceramics, which are often difficult to join using fusion-based processes due to their high melting points, low thermal conductivity, and susceptibility to cracking. Therefore, by avoiding the liquid–solid phase transition, these solid-state techniques can be valuable in preventing the formation of undesired phases, distortions, and high residual stresses, which are common welding defects. Additionally, the microstructural evolution at the joint interface is influenced by the ceramic’s response to temperature and pressure, particularly within the heat-affected zone (HAZ), where grain growth and microcracking are prone to occur, depending on the material’s inherent thermal and mechanical properties [19,20,21].

Another effective technique to obtain similar and dissimilar joints in ceramics is via brazing [22,23]. This technique involves introducing a metallic filler with a lower melting point between the BMs, which remains solid during the procedure, where it is drawn in by capillary action and forms a bond between them. In this case, joints typically exhibit three distinct regions: the fusion zone (FZ), where melting and solidification occur, the HAZ, which experiences elevated temperatures, causing microstructural changes without melting, and the BM, which remains unaffected by the thermal cycle. However, ceramics pose specific challenges due to their poor wettability compared to conventional alloys and significant differences in thermal expansion compared to metals. Such a mismatch can lead to the development of residual and thermal stresses during cooling, which are a primary cause of joint failure. To address these issues, active brazing is often used, where reactive elements are added to the filler, to improve wetting and promote strong chemical bonding with the ceramic, thus resulting in more reliable joints [22,24].

From a slightly different perspective, fusion welding processes involve the melting of the BMs to be joined, followed by solidification to form a continuous joint. While such an approach is well established for metals, its application to ceramics is highly limited [17]. When applied to ceramics, fusion welding can lead to the degradation of mechanical performance. This is mainly due to ceramics’ low fracture toughness, high brittleness, and sensitivity to thermal gradients, which can cause thermal cracking, the development of high residual stresses, and the consequent formation of brittle phases upon cooling. Moreover, the chemical incompatibility between different ceramics, or between ceramics and metals, can also yield the formation of undesirable compounds at the interface during mixing in the liquid state. Fusion-based welding techniques include a wide array of processes, such as laser or electron beam welding; nevertheless, arc welding stands as a promising candidate, as it is one of the most prevalent techniques used in the industry.

In this regard, arc welding is commonly employed for joining metals, due to its efficiency, automation, and low operational cost compared to more complex joining techniques [25]. Therefore, its integration into ceramic components can also be of great value, given its widespread availability. Moreover, especially regarding solid-state techniques, arc welding maneuverability facilitates manufacturing or repair operations in ceramic parts. This is particularly relevant for advantageous for large-scale or field applications, where other joining techniques cannot meet the specific requirements of limited access, irregular geometries, cost constraints, or the need for rapid on-site intervention.

For such applications, however, an understanding of the solidification path undertaken by the melting pool via phase diagrams is crucial in fusion welding of ceramics, as they provide critical information on phase stability, solidus and liquidus temperatures, and the phase transformations that occur during solidification and consequent cooling. Such knowledge helps in optimizing welding conditions by aiding in the process of selecting appropriate materials and processing temperatures to simulate and predict microstructural changes and the formation of undesirable phases, which can lead to premature failure of the weld [26,27,28]. As such, in this paper, we present the state of the art on the topic of arc welding of ceramics, covering studies from 2014 onward, thus providing a comprehensive overview of recent advancements and ongoing research trends in ceramics welding. We aim to provide a discussion centered on process parameters, challenges, and performance outcomes associated with arc welding of ceramics, highlighting their potential advantages and limitations.

## 2. Arc-Based Techniques for Welding Ceramic Materials

As has been established, ceramic materials are indispensable in modern industry for their exceptional properties and versatility, and the possibility of using arc welding techniques in ceramic-based components is most relevant. As such, while arc welding has long been a primary method for joining metals, it was historically not applied to ceramics because of an insufficient understanding of their thermal and mechanical behavior under arc conditions. That is, ceramics tend to present significant challenges when subjected to conventional arc welding processes due to their brittleness and high sensitivity to thermal stresses [29]. Moreover, arc-based welding techniques fundamentally depend on electrical current, implying that the ceramic involved in the process exhibits sufficient electrical conductivity to sustain the electric arc. However, modern developments now enable the successful welding of similar ceramics and even dissimilar joints between ceramics and metals. That is, despite the technical difficulties, ongoing research in ceramic welding contributes to material innovation and the development of more durable, efficient, and lightweight components in various high-tech industries. In fact, in 2011, arc welding of ZrB_2_ was patented by Hilmas et al. [30], as a possibility to form a reliable joint under a controlled non-oxidizing atmosphere.

Therefore, with a focus on establishing the state-of-the-art on arc welding of ceramic materials, the Scopus database was probed to acquire relevant knowledge of the ongoing research on the topic. After filtering the results in accordance with pertinence to the topic, a list of studies relevant to the field is presented in Table 1. From these data, it can be noted that arc welding of ceramics is a pathway to obtain sturdy joints via gas tungsten arc welding (GTAW) and plasma arc welding (PAW). Other options for arc welding would include gas metal arc welding (GMAW) or submerged arc welding (SAW), although in these cases, the filler material would arc as an electrode, which would introduce unnecessary difficulties in managing the welding setup due to the ceramic’s inherent brittleness. Such challenges may include higher deposition rates, less control of the heat input, and arc stability at the joint interface. These challenges are especially problematic for brittle ceramic materials, where precise thermal management and a stable, contamination-free arc are essential to minimize cracking and defects.

While both GTAW and PAW processes are well-established arc welding processes based on the use of a non-consumable tungsten electrode and an inert shielding gas, their operational characteristics and performance differ significantly. Nevertheless, both have been successfully applied to ceramics, and the choice between processes may rely on project specifications.

**Figure 1:**
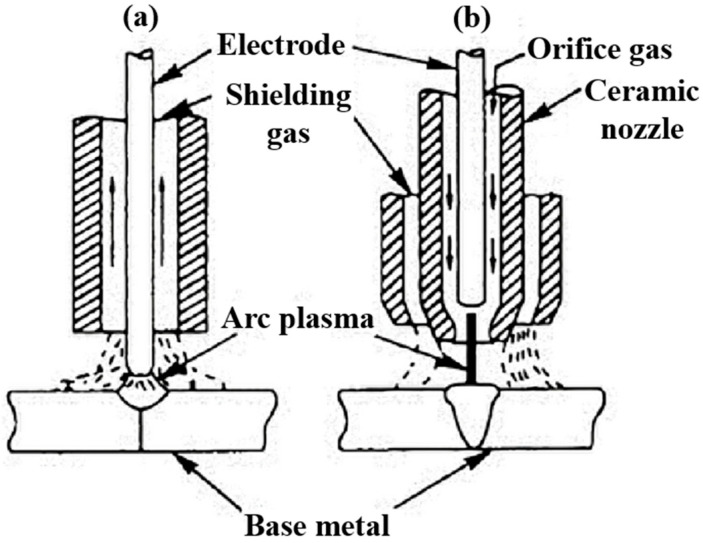
Representative schematic of (**a**) GTAW and (**b**) PAW processes (adapted from [32]).

Represented in Figure 1a, GTAW, also known as Tungsten Inert Gas (TIG) welding, is a fusion welding process capable of producing high-quality bonds between metallic components. This method relies on an electric arc established between a non-consumable tungsten electrode and the workpiece. The tungsten electrode, which protrudes from the torch, can withstand these extreme conditions with minimal erosion due to its high melting point of approximately 3410 °C and excellent thermal stability [31]. Additionally, to protect both the molten pool and the electrode from oxidation and allow the electric arc to form, an inert shielding gas, such as Argon, is applied to the molten pool through the torch. As such, GTAW is valued for its ability to produce precise, clean welds with low spatter and minimal distortion, making it widely used in applications demanding high-quality joints. However, the process typically requires greater operator skill, which is a challenge that can be overcome using computerized numeric control equipment, has slower deposition rates compared to consumable-electrode methods, and is, therefore, more suited to thin sections and critical joints where quality outweighs productivity [25].

On the other hand, PAW (refer to Figure 1b) is also a viable option for joining ceramic materials, as it operates on principles similar to GTAW, by employing a non-consumable tungsten electrode and an electric arc to generate the necessary heat for welding. However, a key distinction lies in the electrode configuration, in which case, the tungsten electrode is fully enclosed inside the welding torch. Such a design constricts and stabilizes the arc as it passes through a fine-bore copper nozzle, transforming it into a high-energy-density plasma jet. With plasma core temperatures exceeding 20,000 °C, this process is capable of producing narrow joints with deeper penetration. Nevertheless, while PAW provides superior control, precision, and penetration, it requires more complex equipment and careful parameter adjustment, which results in higher costs, which often limit its use to critical engineering applications [33].

**Table 1:** Summary and description of peer-reviewed publications regarding arc welding of ceramic materials.

Ceramic System	Arc Welding Technique	Comments	Ref.
ZrB_2_–SiC and ZrB_2_–SiC–B_4_C–YAG	GTAW (with ZrB_2_–SiC–B_4_C–YAG as filler material)	Increase in microhardness across the joint was noticed due to increase in volume fraction of SiC and B_4_C. No evidence of cracks was identified in the ZrB_2_–SiC–B_4_C–YAG joint. Manual welding was conducted.	[34]
SiC-ZrB_2_-ZrC	GTAW	A minimum ZrB_2_ content of 20 vol% is required for welding of SiC-ZrB_2_-ZrC ceramics due to narrow range of weldable compositions that form ZrC as a primary phase.	[35]
SiC-ZrB_2_-ZrC	GTAW	Different compositions were welded using distinct techniques, and the mechanical performance of the joints was assessed at different temperatures.	[36]
SiC-ZrB_2_	PAW		
TiB_2_-TiC	PAW	Successful welding of ceramics was noted, yet large crystals and pores developed within the FZ, which was hinted to hinder the mechanical performance of the welded joint.	[37]
ZrB_2_-ZrC	PAW	Full-penetration joints were obtained with the added variant of using a graphite support, which causes carbon to diffuse to the FZ. The presence of pores was evidenced at the boundary between the FZ and the HAZ.	[38]
ZrB_2_-ZrC and ZrC–TiC	PAW	Mechanical performance was assessed in order to obtain optimal welding conditions/parameters.	[39]
SiC–ZrB_2_–ZrC	PAW	Optimization of welding parameters took place, and the solidification path of the FZ was identified. SiC content varied across the FZ, decreasing from the fusion boundary toward the center.	[40]
ZrB_2_-SiC-ZrC and Mo-based metals	PAW (Dissimilar arc welding)	Dissimilar welding between ceramic and metal was successfully conducted, although defects were unavoidable.	[28]

### 2.1. Gas Tungsten Arc Welding of Ceramics

Focusing on GTAW, Krishnarao et al. [34] conducted GTAW in hot-pressed ZrB_2_–SiC and sintered ZrB_2_–SiC–B_4_C–YAG composites with and without a filler material composed of ZrB_2_–SiC–B_4_C–YAG. The experiments were conducted with a current ranging from 90 to 120 A at a manual welding speed of 3 mm/min, without preheating, post-controlled cooling, or shielding gas. During pre-tests without filler, material cracks and non-uniform joints were obtained, whereas, when filler was employed, coherent joints without cracks were obtained in the ZrB_2_–SiC–B_4_C–YAG case. This resulted in an increase in microhardness across the weld interface, which was related to a higher amount of SiC and B_4_C on the joint stemming from the filler material.

Overall, the study presents a proof-of-concept that GTAW can be a viable option to join ceramics, especially in relation to the necessity for applying a filler material with the same composition as the BM to avoid welding defects. Nevertheless, significant cracking was noted to occur in the ZrB_2_–SiC case, which rendered the joint unusable. Considering the manual processing conditions without the application of shielding gas or preheating procedures, it is remarkable that a welded joint was successfully obtained in the ZrB_2_–SiC–B_4_C–YAG case, which emphasizes its excellent weldability. However, the lack of subsequent information on the matter makes it difficult to attest to the feasibility of GTAW on this ceramic material and whether the procedure can be repeated, especially when manual welding was conducted. Alongside this, provided that shielding gas is a significant factor in GTAW when striking and maintaining the electric arc, this is a line of research that should be further followed in order to make the process stable and reproducible. Nevertheless, as suggested, complementary solutions to cracks may be addressed via preheating and a controlled atmosphere.

**Figure 2:**
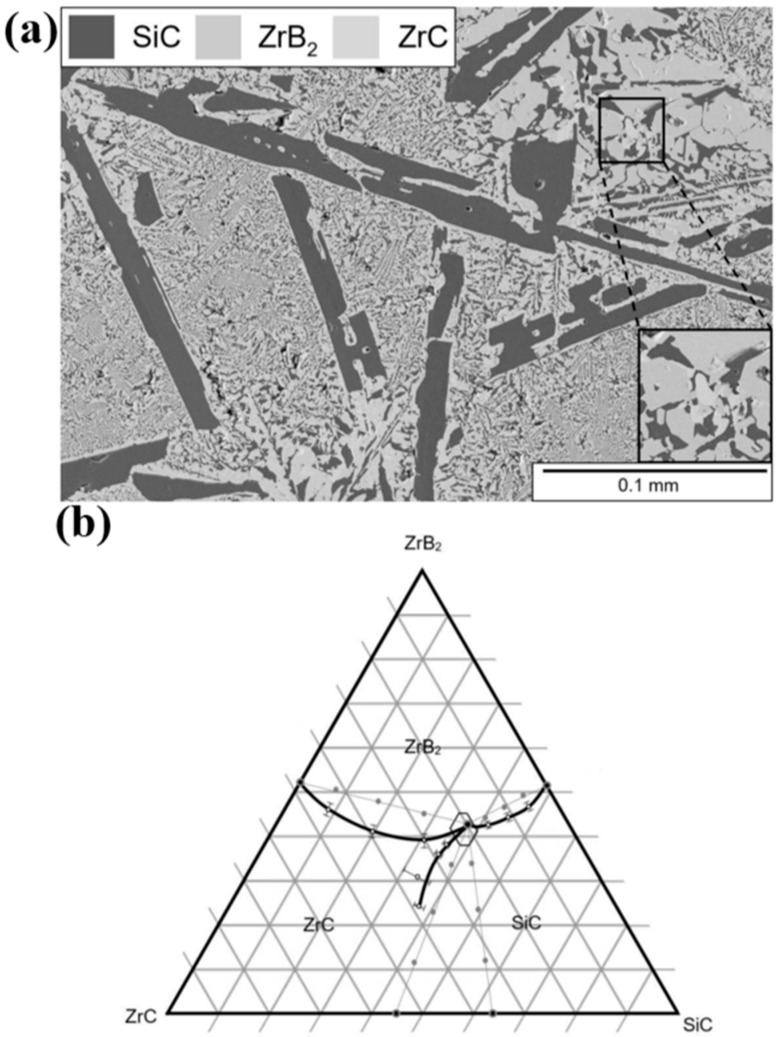
(**a**) Secondary electron micrograph of weld microstructure and (**b**) proposed phase diagram for the SiC-ZrB_2_-ZrC system. Adapted from [35].

Subsequently, Jarman et al. [35] studied the GTAW process in a similar ceramic system, SiC-ZrB_2_-ZrC, with a focus on relating SiC content to its weldability and microstructure. For such, ceramic samples with varying compositions were produced from commercial powders via hot pressing. Prior to welding, the samples were preheated, and full penetration was obtained with the amperage varying between 160 and 200 A and a torch travel speed of 4 cm/min. Overall weldability was noted to improve as compositions approached the ternary eutectic composition, which was identified at 36.9 ± 1.3 vol% SiC, 42.7 ± 1.5 vol% ZrB_2_, and 20.4 ± 1.9 vol% ZrC, corresponding to a melting point of 2330 ± 23 °C. Nevertheless, optimal compositions for welding showcased a SiC content ranging from approximately 36 to 57.5 vol%, which resulted in smaller SiC grain sizes at the joint. Moreover, microstructural analysis permitted the development of a ternary phase diagram, albeit including incomplete phase boundaries between the ternary eutectic and the ZrC-SiC binary compositions (refer to Figure 2). This was due to gas formation related to a low ZrB_2_ content, which allowed us to conclude that a minimum of 20 vol% ZrB_2_ was necessary to ensure good weldability on the SiC-ZrB_2_-ZrC system.

From a different perspective on the experimental approach, it can be noted that the authors used thoughtful research analysis to explore and determine the optimal GTAW process parameters and compositions of the SiC-ZrB_2_-ZrC system. An outstanding effort in constructing phase diagrams was demonstrated, providing a clear visual representation of the relationships between composition, temperature, and phase stability. Such an approach allows not only the identification of phases but also a direct correlation between microstructure and material behavior. This level of detail is particularly valuable, providing a solid foundation for understanding weldability and serving as an indispensable tool to guide both synthesis and processing of the SiC-ZrB_2_-ZrC ceramic. Nevertheless, while ensuring a deep microstructural understanding of the joints, the absence of a detailed mechanical property evaluation leaves the long-term structural performance unclear.

This, however, was addressed in a follow-up study [36], where the authors investigated the mechanical properties at different temperatures of arc-welded SiC-ZrB_2_ and SiC-ZrB_2_-ZrC ternary eutectic ceramic joints obtained via GTAW and PAW, depending on the composition, since PAW is also a viable option for arc welding ceramic materials. Nevertheless, with a focus on the GTAW process, the welding process was once again validated on the SiC-ZrB_2_-ZrC ceramic, allowing for a combination of the microstructural insights with mechanical performance data at high temperatures. As such, similarly, samples were obtained via hot pressing commercial powders, machined and preheated to 1600 °C prior to welding. Worthy of note, upon four-point bending testing at room temperature, the flexure strength of the SiC-ZrB_2_-ZrC joints achieved 258  ±  36 MPa, retaining 38% of the flexure strength of the BM. With increasing temperature, however, such values tended to decrease, achieving 171  ±  30 MPa, at 1700 °C, which corresponds to roughly 59% of the values achieved by the BM at the same temperature. This was attributed to the ZrC content, which reduced the melting point of the material and contributed to the reduction in pores in the process, which was concluded to be the limiting factor in the mechanical performance of the joints. The presence of pores was, nonetheless, a limiting factor in the mechanical performance of all welds.

From these studies, a collective analysis that allows us to trace a clear progression from feasibility and microstructural understanding to functional performance evaluation can be noted.

Such a line of research provides a comprehensive picture of how composition, welding parameter optimization, and microstructural features can influence the integrity and reliability of ceramics processed via GTAW, therefore permitting evaluation and optimization of these materials, which can be reliable in high-temperature applications.

Subsequently, building on these insights from GTAW-processed ceramics, which remain relatively underdeveloped, naturally leads to the exploration of more advanced joining techniques, namely PAW, which can offer greater control over heat input and microstructure.

### 2.2. Plasma Arc Welding of Ceramics

**Figure 3:**
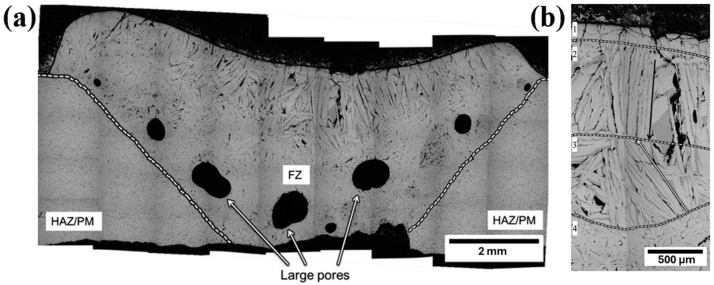
Cross section of a TiB_2_–20TiC-welded joint (**a**) Macrography and (**b**) detailed view highlighting the directional growth of TiB_2_ crystals. Adapted from [37].

Regarding PAW, the TiB_2_-TiC ceramic system was researched by King et al. [37], using a set of parameters comprising a plasma flow rate of 0.75 L/min, a current of 135 A, and a torch speed of 8 cm/min. The procedure also included a preheating step, to approximately 1450 °C, which resulted in the full-penetration joint displayed in Figure 3.

Aside from evident pores on the bottom of the FZ and cracks at its top, which can act to the detriment of mechanical performance, different microstructural regions were visible, highlighting the different conditions that affect the solidification of the joint. Such regions are showcased in Figure 3b, indicating that, during solidification of the molten pool, distinct crystal growth and nucleation phenomena occurred. There, it was noted that the top and bottom of the FZ exhibited smaller TiB_2_ crystals, attributed to higher nucleation rates, whereas the middle of the FZ showed higher growth rates, with TiB_2_ crystals growing up to 1.2 mm in length.

While a deeper discussion on the variation in solidification parameters (growth rate and thermal gradient), with the distance from the torch, and a consequent mechanical performance analysis, would strengthen the discussion, the deep microstructural insight into the structures that developed upon cooling is invaluable. Moreover, it is also interesting to point out that this was the only study that addressed a ceramic system that does not contain ZrB_2_, instead opting to conduct research on a TiB2-based system, which hints at the multiple ceramic possibilities for welding in high-temperature resistant ceramic applications.

A similar approach was taken by the same research group with ZrB_2_–20 vol% ZrC ceramics welded using PAW. The procedure was achieved using a current of 198 A, plasma flow rate of 0.75 L/min, and welding speed of approximately 8 cm/min, with the added variant of welding with and without the aid of a graphite support [38].

Depending on the welding setup, the obtained FZ differed in terms of microstructure, which, in the case welded without the graphite support, showcased a coarse microstructure with elongated ZrB_2_ grains, embedded in a ZrB_2_–ZrC eutectic, containing approximately 43 vol% ZrC. On the other hand, the joints obtained with the aid of the graphite support exhibited finer and asymmetric grains of ZrB_2_ and ZrC, alongside carbon, which diffused from the graphite support. This indicates that, at the high temperatures achieved with PAW, carbon atoms from the graphite diffused into the molten ceramic, which led to localized carbon enrichment in the FZ. As a result, the weld exhibited an unintended change in composition and a non-uniform microstructure, making it less consistent and predictable. Additionally, the presence of porosities within the joints was also noted to occur in both cases.

However, subsequent mechanical testing indicated that, in both cases, the FZ has a lower hardness than the heat-affected zones, which in turn, were also lower than the base material. Nevertheless, the main takeaway is that adding a platform to improve the practicability of PAW can be a viable option, but the prospect that graphite remains inert and does not interfere chemically with the weld is not. Therefore, non-carbon supports or protective barriers should be preferred to avoid contamination of the joints.

In complement to this investigation, the relation between mechanical properties and the nucleation of flaws during welding was also mentioned in a subsequent study [39], where the mechanical performance of ZrB_2_–ZrC and ZrC–TiC ceramics was analyzed and compared. In this study, the PAW parameters were selected based on each ceramic system, coupled with a preheating stage was conducted. Overall, the welded specimens evidenced a reduced mechanical performance when compared to each BM, which was attributed to the amount of porosities, microcracks, and the large ZrB_2_ grains contained within the solidified molten pool, thus emphasizing the importance of controlling such solidification-related issues. As such, although highlighting the versatility of PAW to fabricate thermocouples, obtain complex shapes, and emphasize the possibility to join ceramics with different compositions, the necessity for future research to control grain size and reduce defects to obtain welded ceramics with improved mechanical properties was accentuated.

**Figure 4:**
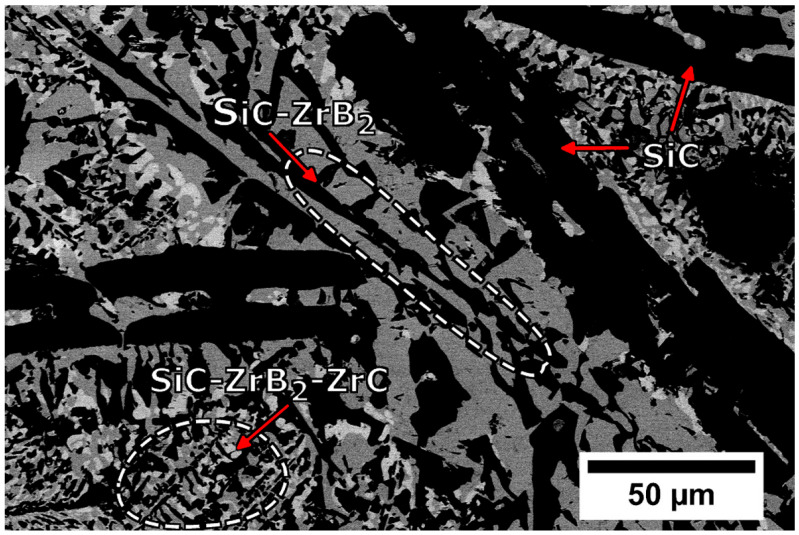
Backscattered electron image obtained at the middle of the FZ. From [40].

On a further note, autogenous welding, via PAW, was conducted by King et al. [40] on a silicon carbide-based ceramic, containing 50 vol% SiC, 35 vol% ZrB_2_, and 15 vol% ZrC. For welding, the BM was preheated to 1450 °C, and PAW was successfully conducted using a current of 138 A, a plasma flow rate varying between 1 and 0.5 L/min, and a welding speed of 8 cm/min. It was observed that full-penetration welds with minimal mass loss (below 1 wt%) were obtained, marking a significant advancement in ceramic joining methods, while offering potential improvements in structural integrity for high-performing ceramics. In terms of microstructural analysis, the solidification path initiated with the precipitation of SiC, followed by the simultaneous solidification of SiC and ZrB_2_, concluding with the formation of a ternary eutectic reaction composed of the three phases SiC, ZrB_2_, and ZrC. Such is evidenced in Figure 4. Furthermore, the molten pool dynamics led to SiC enrichment at the edges of the FZ, though the overall nominal SiC content remained close to that of the parent material.

Although we are not delving into the analysis of mechanical properties for the SiC-ZrB_2_-ZrC system, a ceramic containing the same components, although with slightly different composition, was welded using GTAW, and its mechanical performance was tested at different temperatures and compared to a PAW-welded SiC-ZrB_2_ system [36]. Provided that inferring the mechanical performance of the PAW-welded SiC-ZrB_2_-ZrC ceramic from this study is not possible, it is still important to mention that introducing ZrC to the SiC-ZrB_2_ ceramics caused the mechanical performance to deteriorate at high temperatures. This, nonetheless, can also be a product of the different methods employed to obtain each type of joint, thus highlighting a research gap that should be further addressed.

**Figure 5:**
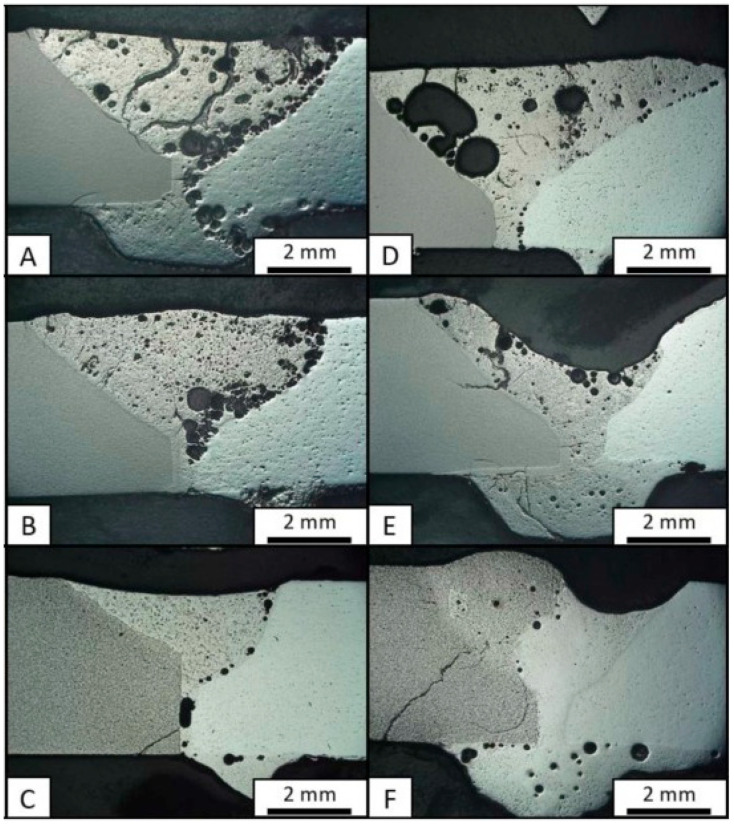
Representative cross sections of dissimilar welded joints obtained between ceramics and metals: (**A**) ternary eutectic composition of the ZrB_2_-SiC-ZrC ceramic system with TZM; (**B**) 70 ZrB_2_ ceramic with TZM; (**C**) pure ZrB_2_ ceramic with TZM; (**D**) ternary eutectic ceramic with Mo; (**E**) 70 ZrB_2_ ceramic with Mo; and (**F**) pure ZrB_2_ with Mo. Note that porosities and cracks are present in all cases. Adapted from [28].

While similar welding focuses on joining the same metals, dissimilar welding deals with combining different metals, bringing its own set of challenges and techniques. Interestingly, this has been researched by Jarman et al. [28]. In this research, the possibility of dissimilar welding of refractory Mo-based metals to ZrB_2_-SiC-ZrC ceramic systems, with varying amounts of ZrB_2_, was conducted. Hence, commercially pure Mo metal and a Mo alloy containing titanium and zirconium additions (TZM) were joined via PAW to the ceramic material, with the intention of evaluating the influence of thermal and electrical properties on metal–ceramic fusion welds. The welding procedure included a shielding gas flow of 7.5 L/min and a plasma flow of 1 L/min, at a travel speed of 13 cm/min. After welding, specimens were covered with graphite felt, heated to 1600 °C, cooled at 10 °C/min to 1350 °C, annealed for one hour, and then cooled at 5 °C/min to room temperature. The resulting joints are presented in Figure 5. Although no discernible effects on weld quality were noted due to changes in the thermal conductivity or electrical resistivity, the overall results showcased that the Mo alloy had better weldability, based on the consistent penetration depth attained in the dissimilar joints. Nevertheless, all metal–ceramic joints contained porosities within their corresponding FZs, whereas it was noted that, in a pure Mo weld, porosity tended to occur at the FZ center, while in the case of the Mo alloy, porosity was mainly located at the boundaries between the ceramics and the FZ interfaces. Nevertheless, it was observed that, with the increasing content of ZrB_2_ within the ceramic material, the number of defects tended to decrease. This study also hinted that other factors, such as viscosity and wettability, must be accounted for in ceramic–metal joints, thus marking a possible onset for future investigations on the topic of arc welding of different classes of materials.

## 3. Discussion and Future Outlook

As mentioned, the use of arc welding techniques for joining ceramics presents a promising pathway for developing high-performing monolithic components from separate ceramic parts.

Provided that the application of arc welding to ceramics remains in its infancy, with only a limited number of studies exploring its feasibility and practical implementation, a deeper understanding of the arc welding process and weld pool mechanics could potentially lead to significant improvements in joining ceramic materials. Nevertheless, the existing literature has already proven that arc welding is possible, by providing a deep microstructure discussion on the obtained welded joints. The topic, however, would benefit from thermodynamically-based simulations, which would allow for the prediction of the phases that are generated upon cooling and determination of solidification paths, and consequent correlation with mechanical performance.

Nevertheless, up to now, progress in research suggests a concerted effort to enhance material performance and overcome the inherent challenges associated with ceramic joining. Of special note is the fact that, among the welded ceramics systems, ZrB_2_ has been the most extensively studied for arc welding applications, whereas TiB_2_ remains largely unexplored, highlighting the need for further investigation into less common ceramic materials. The choice of such systems, however, requires ceramics to exhibit an electrical conductivity similar to metals, which is a necessary requirement to strike and maintain the electric arc required to achieve high temperatures for welding.

Moreover, joining ceramics via arc welding techniques also presents other significant challenges that hinder its widespread adoption. One of the primary difficulties arises from low thermal conductivity and high thermal expansion, which can lead to thermal stresses and cracking during the rapid heating and cooling cycles inherent to arc welding-based processes. Additionally, the brittle nature of ceramic materials makes them highly susceptible to fracture, as they cannot undergo plastic deformation to relieve internal stresses during cooling and consequent solidification. Such phenomena can be aggravated by the fact that ceramic materials, due to their high temperature stability, may not melt uniformly, thus complicating the fusion process, resulting in the development of porosities, incomplete fusion, and cracking, thus hindering the integrity of the joint. On the other hand, material compatibility is another concern, as not all ceramics respond similarly to arc welding, particularly in dissimilar material joints, provided that pores and unbonded regions at the metal–ceramic interface can increase stress concentration and act as initiation sites for cracks.

The equipment and setup required for ceramic arc welding, either via GTAW or PAW, can be complex, requiring specialized handling when considering the high temperatures required for preheating, which can decrease its cost-effectiveness in practical terms. Collectively, these technical and economic challenges contribute to the limited industrial interest and slow progress in developing arc welding as a reliable method for joining ceramic components.

Nevertheless, the choice between these two processes relies on the specific requirements of the ceramic material being joined, including its thermal sensitivity, its mechanical properties, and the geometry of the components. In this regard, PAW can be favored when greater precision and deeper penetration are required; however, due to its higher sensitivity to process parameters, GTAW can be a better solution for cases where a simpler setup and better accessibility are essential. Ultimately, the selection depends on balancing joint quality, process complexity, and the ceramic’s tolerance to the welding thermal cycles, although other ceramic bonding techniques can be employed, such as laser welding or solid-state bonding. Nonetheless, arc welding offers a viable route for ceramic welding operations, provided it is properly optimized through preheating procedures and/or the use of suitable filler materials. Its potential advantages in terms of adaptability and accessibility must, nonetheless, be further researched via cost and process-efficiency analyses.

Looking forward, the future of arc welding for ceramics appears promising, albeit contingent on addressing the current technical and material challenges. Advancements in process modeling and the expansion of research beyond well-studied systems, while studying the mechanical behavior of the arc-welded joints, may enable reliable joint design and offer practical manufacturing solutions. As such, with sustained research efforts and technological refinement, arc welding has the potential to evolve into a versatile and industrially viable method for joining ceramics, bridging the gap between high-performance material requirements and ceramic materials.

## 4. Concluding Remarks

In conclusion, despite the scarcity of existing studies, the application of GTAW and PAW in ceramic processing represents a promising avenue for further research, as it opens the pathway to new engineering solutions by creating welded joints between ceramics or ceramics and metals. Hence, the ability to create reliable and durable joints between such distinct classes of materials is something of great interest to sectors such as aerospace, biomedical, and electronics, where such welded joints are highly desirable.

Moreover, current results indicate that, while arc-based approaches can achieve joints with strengths in the range of 140~258 MPa [36,39], systematic investigations into the mechanical properties of such joints remain very limited. Additionally, these values are still generally associated with the existence of welding defects, such as porosities, and the fact that preheating the materials to be welded can be a challenging task. However, further research to adjust the heat input and strengthen the precision of these welding methods can provide the means to overcome such limitations in ceramics joining, especially when combined with innovative approaches and strict control of welding parameters.

Nevertheless, the ability of GTAW and PAW to operate with relatively simple equipment, lower material costs, and adaptability to complex geometries makes them an attractive area for further study. However, for industries such as aerospace, biomedical, and electronics, even incremental improvements in arc-based ceramic joining could provide accessible alternatives where high-temperature or vacuum-based techniques are impractical.

Therefore, arc welding emerges as a relevant and strategic research topic for the future of ceramics processing. As a result, investing in further experimental and theoretical studies in this area could not only fill a scientific gap but also stimulate the development of such welding technologies for high-performing applications.

## Data Availability

No new data were created or analyzed in this study. Data sharing is not applicable to this article.
